# Three-Dimensional Porous Media Design and Validation for Fluid Flow Applications in Hydrocarbon Reservoirs

**DOI:** 10.3390/mi17040430

**Published:** 2026-03-31

**Authors:** Omer A. Omer, Khaled S. Al-Salem, Zeyad Almutairi

**Affiliations:** Mechanical Engineering Department, College of Engineering, King Saud University, P.O. Box 800, Riyadh 11421, Saudi Arabia; kalsalem@ksu.edu.sa (K.S.A.-S.); zaalmutairi@ksu.edu.sa (Z.A.)

**Keywords:** porous media, porosity, permeability, three-dimensional micromodel, three-dimensional computer-aided design modeling, fluid flow, hydrocarbon reservoir

## Abstract

This study introduces a computational method for designing realistic, geometrically controlled three-dimensional (3-D) micromodels of porous media to investigate fluid flow in hydrocarbon reservoirs. The methodology utilizes a virtual framework of cubes where an arbitrary, continuous 3-D pore network is generated via two-dimensional (2-D) sketches. A key strength of this deterministic, cube-by-cube approach is the ability to independently control porosity and permeability by adjusting channel size and connectivity, facilitating the systematic study of spatial heterogeneity. Six digital models were developed with porosities ranging from 18.4% to 44.4%. Unlike traditional stochastic algorithms, this explicit geometric control enabled the accurate extraction of pore volume distributions and the establishment of a robust power-law relationship between localized porosity and specific surface area. Statistical analysis confirmed a linear correlation between porosity and pore dimensions. While focusing on design and validation, these models are 3-D printable and provide exact boundary conditions for CFD simulations. Single-phase simulations confirmed the capability to decouple absolute permeability from porosity. Consequently, this framework bridges the gap between numerical simulations and physical laboratory experiments to optimize Enhanced Oil Recovery (EOR) processes.

## 1. Introduction

The simultaneous motion of crude oil, natural gas, formation water, and injection water containing dissolved chemicals through hydrocarbon reservoir rocks represents one of the most complex forms of multiphase flow in porous media. Each of these fluids behaves as a distinct phase with unique properties, since they do not form a homogeneous solution with one another, particularly in the case of formation water or water-based solutions with oil and gas.

A reservoir rock is a subsurface volume of rock, composed of a solid mineral matrix, which features a complex, three-dimensional network of interconnected and isolated pores. This specific architecture provides the rock with sufficient porosity to store hydrocarbons and sufficient permeability to both transmit migrating fluids and allow for their accumulation under appropriate trap circumstances [1]. Porosity measures the total volume of empty space within a rock, which is made up of a complex network of pores. Its value is determined by the amount and size of these pores. Essentially, it is the rock’s ability to contain fluids. In contrast, permeability measures how easily fluids can flow through the rock. It is not just about how much empty space there is, but about how well-connected those pores are. A rock can have a lot of pores (high porosity) but still have low permeability if the pores are not connected. Both the amount and size of the pores, along with their degree of connection, determine the permeability. A fundamental understanding of multiphase flow physics at the pore scale is critical for optimizing EOR strategies. As highlighted by Blunt [2], the complex interplay between capillary forces, wettability, and pore-scale geometry governs the mechanisms of fluid trapping, and therefore dictates the efficiency of any recovery operation. Pore-scale modeling provides an indispensable link between microscopic properties (e.g., pore geometry, wettability) and the macroscopic displacement mechanisms relevant to EOR. As reviewed by Golparvar et al. [3], this approach is crucial for accurately determining the core flow functions used in continuum-scale models to predict recovery efficiency.

**Figure 1:**
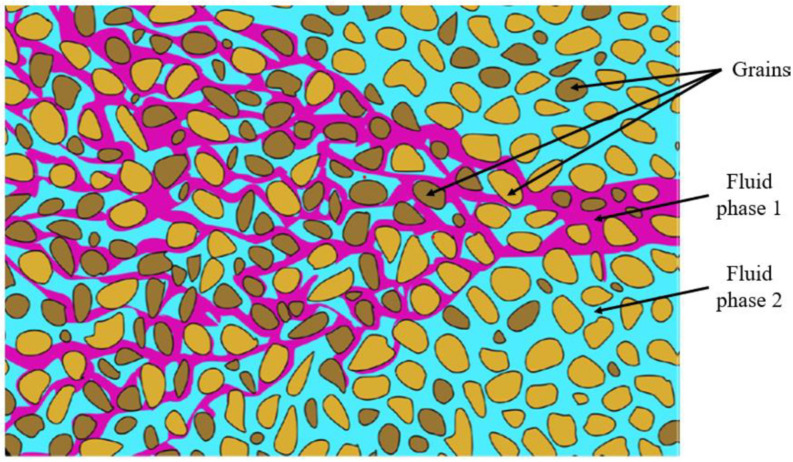
Two-phase flow in a porous medium [5].

The behavior of these coexisting fluids is governed by the fundamental principles of fluid mechanics, specifically the conservation laws. These laws of mass, momentum, and energy provide the scientific framework for understanding and predicting how these different fluids move and interact through the porous rock. Essentially, they ensure that the total mass, force, and energy of the system are accounted for as the fluids travel from the reservoir to the production wells [4]. Figure 1 illustrates a basic configuration of two-phase fluid flowing through a porous medium [5].

The complexity of pore geometries and the heterogeneity of a porous medium cause the macroscopic behavior of fluids to be difficult to predict at the pore level [6]. The oil and gas industry depends extensively on laboratory core flooding experiments to evaluate new oil recovery techniques for specific geological formations. This approach at laboratory scale is crucial for assessing the applicability of new flooding methods without resorting to expensive field drilling. In these experiments, an injection fluid is pumped through a core sample that mimics the reservoir rock, typically an unconsolidated pack of sand grains [7,8,9,10] or a consolidated core of sandstone or carbonate [11,12,13,14]. The effectiveness of the technique is then determined by measuring the resulting oil recovery [15].

**Figure 2:**
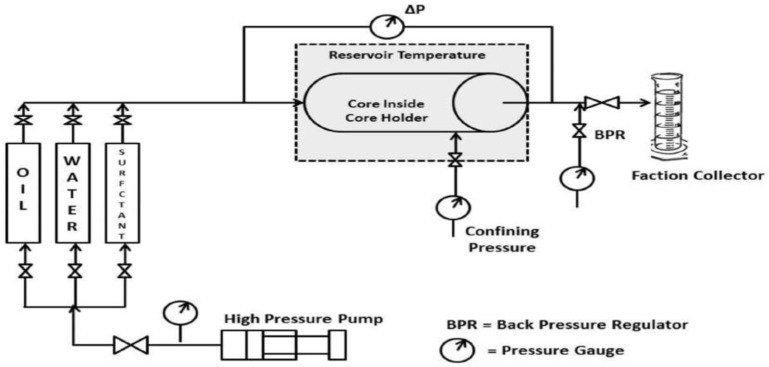
A typical core flooding unit used to surfactants flooding [16].

Figure 2 illustrates a typical core flooding apparatus for surfactant flooding experiments, similar to the one used by Alameri et al. [16,17]. The setup comprises three main sections [15]: an upstream section, a core block, and a downstream section. The upstream part is responsible for delivering fluids to the core block via pumps and accumulators. The core block, which holds the porous medium, is designed to simulate reservoir conditions by applying overburden pressure and using a thermal jacket to control temperature. Finally, the downstream section is used to collect the effluent from the core with fraction collectors or phase separators. Conventional core-flooding analysis, while standard, suffers from significant intrinsic limitations that justify the use of micromodels [18]. The primary drawback [18] is the opaqueness of natural rock, which prevents direct visualization of pore-scale fluid dynamics, effectively treating the sample as a “black box.” This issue is exacerbated by the uncontrolled heterogeneity in pore geometry and mineralogy, making it difficult to isolate specific physical parameters. This inherent complexity and uniqueness also lead to significant challenges in experimental reproducibility, as no two cores are identical. Furthermore, these traditional methods are notoriously time-intensive and costly, slowing research cycles and limiting parametric investigation.

Over the past decade, researchers have increasingly adopted transparent microfluidic models, commonly known as micromodels, to simulate porous media [19]. These apparatuses feature a transparent, interconnected porous network that enables the direct visualization of complex internal fluid flow dynamics [20]. Due to the opacity of typical three-dimensional media, macroscopic transport measurements cannot provide visual access to flow behavior. As a result, the specific conditions under which these transport mechanisms happen are unknown. Flow imaging in micromodel systems is therefore crucial for providing key insights into the nature of the flow [21].

The necessity of observing the pore-scale interactions is exemplified by the work of Jain et al. [22], who investigated the critical relationship between wettability and fluid-fluid interfacial area using an unconsolidated porous medium of glass beads. This is a practical application of the fundamental principles of fluid mechanics and capillary forces mentioned previously. By comparing a baseline water-wet system to a treated oil-wet system under low-rate, capillary-dominated flow, they observed a significant difference in how the interfacial area behaved relative to saturation. While the water-wet medium showed only a slow increase in interfacial area as water saturation decreased, the oil-wet medium exhibited a distinct trend: the area first increased, reached a clear maximum, and then declined as the system approached residual saturation. This observation is particularly significant as it provides strong experimental validation for existing theoretical models that had predicted the existence of this maximum peak for interfacial area during a drainage process.

Recent comprehensive reviews emphasize that accurately predicting these complex processes requires advanced multiscale modeling approaches capable of capturing the extreme heterogeneity of geological formations [23,24]. At the fundamental level, pore-scale simulations have proven essential for elucidating how specific geometric features—such as grain shape, packing patterns, and boundary conditions—directly govern dynamic fluid displacement mechanisms like spontaneous imbibition [25]. However, conducting such high-fidelity numerical simulations relies heavily on the quality, realism, and geometric controllability of the underlying physical or digital porous media models.

Current pore-scale studies, exemplified by work examining the impact of wettability on fluid displacement [22], overwhelmingly utilize 2-D micromodels or simplified 3-D geometries [20]. Standard fabrication techniques like glass etching and soft lithography (polydimethylsiloxane-PDMS) are inherently limited, predominantly producing 2-D networks [26]. However, the reliance on 2-D architectures represents a significant research gap because these models inherently fail to capture the complex, true-to-life tortuous paths, enhanced connectivity, and pore-to-pore trapping physics characteristic of a real three-dimensional porous network [20,27]. Attempts to create controlled 3-D replicas, such as bead packs or early-generation 3-D-printed models, often introduce new challenges related to visualization (due to refractive index mismatch or limited optical access) or uncontrolled internal geometry and surface quality [20,28]. The present need is therefore focused on developing highly controlled, reproducible 3-D models that accurately reflect geological complexity without sacrificing the necessary optical accessibility for direct numerical simulation [28].

Furthermore, while advanced models reconstructed from X-ray Micro-Computerized Tomography (CT) scans of natural rocks accurately capture true geological complexity, their extreme heterogeneity makes it exceptionally difficult to systematically isolate and control specific geometric parameters (e.g., precise pore-throat sizing or tortuosity) [20,29]. Similarly, algorithmically generated or stochastic pore network models inherently lack the deterministic, localized control required to explicitly design individual pore junctions and pathways [30]. The present need is therefore focused on developing highly controlled, reproducible 3-D models that bridge this gap.

The present need is therefore focused on developing highly controlled, reproducible 3-D models that bridge this research gap. To address these prevalent limitations, the overarching goal of this study is to introduce a novel, fully deterministic CAD-based approach for constructing 3-D micromodels. This versatile computational method provides absolute geometric control over the internal pore architecture, successfully generating realistic porous media that accurately mimic the tortuous paths and geological complexity of actual reservoirs. While this framework can be adapted to generate diverse networks for various applications, its primary objective in this paper is to provide high-fidelity models specifically designed for investigating multiphase flow dynamics within reservoir rocks, particularly during EOR processes. Consequently, the generated models facilitate clear visualization and precise numerical modeling, making them highly suitable for both advanced CFD simulations and 3-D-printable fabrication for laboratory experiments. Finally, this paper outlines the design methodology, presents the geometric analysis of the generated models, and concludes with a discussion of their potential future utility in physical and numerical experimentation.

While open-source computational libraries currently exist that can rapidly generate porous media with controlled macroscopic characteristics (such as overall porosity and pore-size distribution), these established tools typically rely on stochastic algorithms. Consequently, they generate statistically equivalent bulk media but inherently lack the capacity for explicit, deterministic spatial control over individual, localized pore and throat morphologies. In contrast, the methodology proposed in this study offers a unique value proposition: exact geometric determinism. By utilizing a manually controlled virtual framework, specific local anomalies—such as precise bottlenecks, sudden expansions, distinct dead-end structures, or exact tortuous paths—can be explicitly engineered at exact spatial coordinates. Furthermore, this proposed method enables the creation of a porous network that explicitly defines the boundary surfaces of the fluid domain. This exact digital representation can be used to fabricate physical 3-D printed micromodels, while simultaneously serving as the precise computational domain for CFD simulations. This seamless integration of deterministic digital design, explicit computational modeling, and manufacturable physical models is a primary advantage that distinguishes the proposed methodology from traditional stochastic methods. Despite this study utilizing single-phase flow simulations to establish the fundamental petrophysical characterization of the designed media, the deterministic 3-D framework is specifically engineered as a high-fidelity platform for future multiphase flow investigations.

## 2. Micromodels of Porous Media Design

The digital micromodels of porous media in this study were generated using a structured methodology executed in three parts. This approach was designed to ensure the accurate and systematic creation of the micromodels. The following provides a detailed description of each stage in the generation process.

1. Network of channels were generated to mimic connected pore channels in hydrocarbon-bearing porous and permeable rock.
2. The inlet bank with a port was created to distribute the injected fluids across the front face of the main porous medium body at a uniform pressure. Similarly, the outlet bank with a port was created to collect the produced fluids from the back face of the main porous medium body at a uniform pressure.
3. Additionally, a main body was constructed to encompass the porous voids, the inlet suction bank, and the outlet discharge bank. The inlet and outlet banks were connected to the inlet and outlet ports, respectively.

### 2.1. Creation of Network of Channels

The proposed main frame of a porous medium micromodel has dimensions of 10 mm in length, 5 mm in width, and 2 mm in height along x-, y- and z-directions, respectively. The primary objective of limiting the model dimensions to this small size is to achieve a Representative Scale (RS) that accurately captures micro-scale fluid behavior while maintaining computational feasibility. In the experimental phase, this size is sufficient to observe precise fluid exchange and interfacial dynamics without the complexity of macro-scale variables. In the subsequent CFD modeling, these dimensions are crucial because increasing the model size leads to an exponential growth in mesh elements (grid size), which demands significantly higher computational power and memory. By utilizing a miniaturized model, the mesh density can be optimized to provide high-resolution results at the microscopic level. This approach ensures that the simulation remains computationally efficient while providing a detailed and accurate representation of the fluid’s physical behavior.

**Figure 3:**
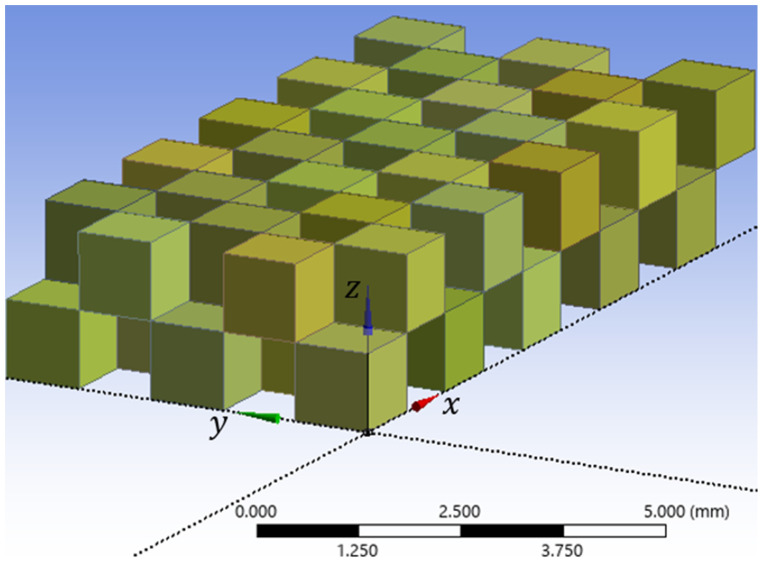
Full frame consisting of 1 mm^3^-voloume fifty cubes in addition to 1 mm^3^-volume fifty imaginary (empty) cubes in a total of 100 cubes.

**Figure 4:**
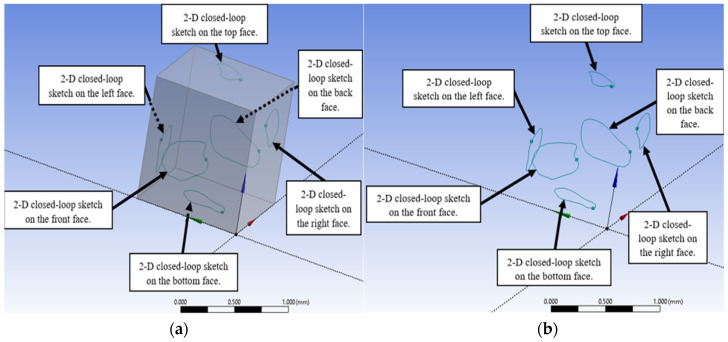
Two-dimensional closed-loop sketches on the faces of the first cube: (**a**) keeping cube to sketch on its faces and (**b**) after cube suppressing.

The graphical design of the porous medium micromodel started with a creation of 50 cubes each having a side length of 1 mm as shown in Figure 3. The geometric modeling started with the establishment of the first micro-cube, which possessed a side length of 1 mm in all dimensions, yielding a volume of 1 mm^3^. Crucially, the front-right-bottom corner of the cube was positioned at the origin of the global coordinates, (0, 0, 0). As illustrated in Figure 4a, this cube is bounded by three pairs of parallel planes: the front and back faces lie on the yz-plane at x = 0 and x = 1 mm, respectively; the right and left faces lie on the xz-plane at y = 0 and y = 1 mm, respectively; and the bottom and top faces lie on the xy-plane at z = 0 and z = 1 mm, respectively.

The next cube (third cube in order) was created with a shifted front-right-bottom corner to global coordinates of (0, 2 mm, 0). Note that a virtual cube separates the first and second created cubes. All subsequent cubes were created in the same way by changing the origin of the generated base plane at (x, y, z) on the global coordinates according to the desired location of the cube as illustrated in Table 1. In this table, the first five cubes and the last five cubes are listed. The full frame containing 50 1 mm^3^-volume cubes is displayed in Figure 3. As shown in this figure, there are 50 imaginary (empty) extra cubes with a side length of 1 mm and a volume of 1 mm^3^. This arrangement is intended to prevent surface duplication at the same plane. This ensures that the generated face of a solid cube serves as a shared boundary for both the adjacent imaginary cube and the next created one, maintaining a continuous and interconnected network of channels which will be created later. The total number of cubes are 100 cubes with a total volume of 100 mm^3^.

These cubes are distributed such that there are 10 rows, each consisting of 10 cubes, oriented in the x-direction over a total length of 10 mm; additionally, there are 5 columns, each consisting of 20 cubes, oriented in the y-direction across a total width of 5 mm; and finally, there are 2 layers (top and bottom), each consisting of 50 cubes, oriented in the z-direction over a total height of 2 mm.

The creation of these cubes is intended to utilize their faces to form three-dimensional pores. The medium consists of an array of cubes, each possessing six faces: front (*f*), back (*b*), right (*r*), left (*l*), bottom (*bo*), and top (*t*). Most of these faces are shared with adjacent cubes. The faces that are not shared are those that constitute the perimeter of the main porous structure, in addition to the faces forming the inlet or outlet of the fluid. Specifically, the front faces of the first ten cubes (*f*_1_ through *f*_10_) will face the suction bank (inlet), while the back faces of the last ten cubes (*b*_91_ through *b*_100_) will face the discharge bank (outlet), both of which are to be created later. The other uncommon faces are situated on the boundaries of the main frame (left, right, bottom, and top sides, such as the bottom faces of the first five cubes *bo*_1_ through *bo*_5_). The remaining faces are shared between adjacent cubes; for instance, the left face of the first cube (*l*_1_*r*_2_) simultaneously represents the right face of its neighboring cube from the left side which is the second cube. This systematic arrangement of cubes and the designation of common and uncommon faces are crucial for forming the required pore network. The second column of Table 1 confirms this interchanging pattern, explicitly detailing whether each cube location is “created” (solid) or “imaginary” (void). The third column (origin of front-right-bottom corner) defines the modular placement of each cube within the overall structure by providing the global coordinates (x, y, z) of its front-right-bottom corner.

In the next stage, the faces of the generated cubes were used as sketching surfaces to create the three-dimensional porous network. For the first cube, arbitrary two-dimensional closed-loop sketches were generated on all six faces, as illustrated in Figure 4a. These sketches were then displayed while the cube structure itself was suppressed, resulting in the foundational geometry shown in Figure 4b.

**Figure 5:**
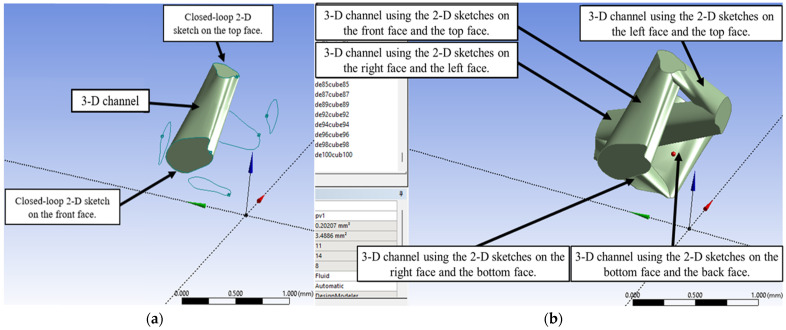
A three-dimensional channel network was generated in the first cube. (**a**) The resulting three-dimensional channel from skin process using the sketches on the front face and the top face of the first cube. (**b**) A total volume of approximately 0.20207 mm^3^ and a total surface area of approximately 3.4886 mm.

To construct three-dimensional channels, the skin process was applied on the generated sketches. For instance, Figure 5a demonstrates the skin process using the sketches on the front face and the top face of the first cube, resulting in the formation of a three-dimensional channel. In a similar way, multiple pores were arbitrarily constructed using the 2-D sketches on the surfaces of the cube as shown in Figure 5b. The resulting porous network occupies a total volume of approximately 0.2021 mm^3^ and has a total surface area of around 3.489 mm^2^. Since the bulk volume of the cube is 1 mm^3^ therefore the porosity in this cube is 0.2021. The sketch on the front face serves as a part of the inlet section, while the sketches on the left face and the bottom face serve as independent boundaries where the channels end. The sketches on the planes on the shared faces (left, back, top) must match the channel structure of their respective neighboring cubes (second, eleventh, sixth).

It is important to emphasize that the finalized pore volume and surface area within the cube were established through an iterative optimization process. This involved multiple design cycles of refining the 2-D sketches on the faces of the cubes and adjusting the skinning trajectories. These trials were repeated until the resulting internal void space aligned with the targeted porosity.

**Figure 6:**
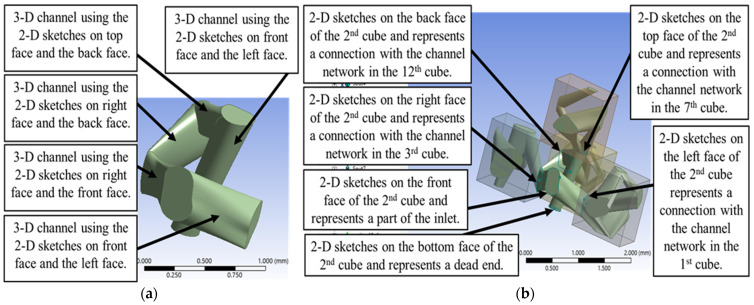
(**a**) A three-dimensional channel network was generated in the second cube, with a total volume of approximately 0.17557 mm^3^ and a total surface area of approximately 3.2621 mm^2^. (**b**) The three-dimensional channel network generated in the second cube and its inlet, dead end and connectivities with the channel networks in the adjacent cubes.

The process for creating the three-dimensional channel network within the subsequent bulk volume, designated as the second (imaginary) cube, was structured to ensure both connectivity and defined boundaries. Planes were generated on the non-shared front face and the bottom face of this imaginary cube (the yz-plane and the xy-plane, respectively). The sketch created on the front face served as a part of the inlet section, while the sketch on the bottom face acted as an independent boundary where new channels could terminate. For the shared faces—the left face, right face, back face, and top face, which connect to the first, third, twelfth, and seventh cubes, respectively—2-D planes and arbitrary 2-D sketches were subsequently created. This technique ensures continuity of the porous network as illustrated in Figure 6b.

Multiple arbitrary channels were then constructed using the skin process on the created sketches. This methodology intentionally introduced variability in pore volume and total surface area between different channel networks, such as those in the first and second cubes. The internal pore network was constructed explicitly manually, without relying on any automated algorithms. Specifically, a single 2-D sketch was manually defined on each shared face between adjacent cubes to serve as a precise transition or junction point for the continuous 3-D fluid channels.

The resulting porous network in the second cube occupied a total volume of approximately 0.1756 mm^3^ and had a total surface area of around 3.262 mm^2^. Given the bulk volume of the cube is 1 mm^3^, the resulting local porosity in this cube was 0.1756. This result is visually shown in Figure 6a. Furthermore, the analysis of the total pore volume of 0.9311 mm^3^ across five cubes (a bulk volume of 5 mm^3^) yielded an average porosity of 0.1862, which falls within the acceptable range of porosity criteria for hydrocarbon reservoir rocks [31].

**Figure 7:**
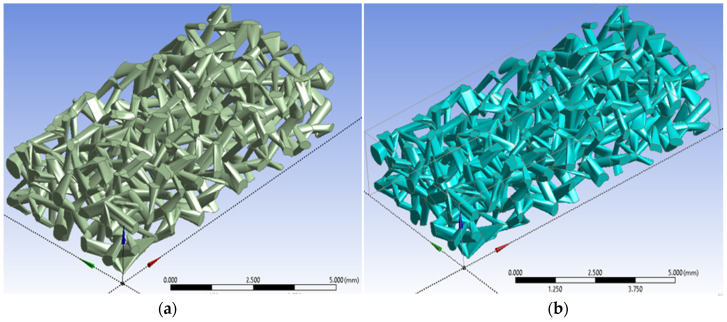
(**a**) The total three-dimensional channel network in all cubes, with a total volume of approximately 18.393 mm^3^ and a total surface area of approximately 299.8 mm^2^. (**b**) The bounding box of the total porous network emphases that the porous micromodel has a length of 10 mm, a width of 5 mm and a height of 2 mm aligned with x-, y- and z-directions, respectively.

The same procedures used to create pore networks in the first and second cubes were applied to all remaining cubes to complete the formation of a connected three-dimensional pore network, as illustrated in Figure 7a. The resulting porous network is enclosed within a bulk volume of 100 mm^3^, which is defined by a rectangular cuboid measuring 10 mm in length, 5 mm in width, and 2 mm in height, as demonstrated in Figure 7b.

The model shown in Figure 7 was created by following all these steps, and this model was denoted as model *A*. The 100-cube-frame that had been created, shown in Figure 3, was copied, then all subsequent steps were reapplied to create five additional models with different porosities and total surface areas. All models were given a bulk volume of 100 mm^3^ since they were generated based on the frame shown in Figure 3. The resulting models are denoted as models *B*, *C*, *D*, *E* and *F*.

As previously mentioned, the generation of the 2-D sketches and the subsequent skinning operations on the faces of each virtual cube to create 3-D channels were executed manually through a trial-and-error process. Although the porous structure was constructed using discrete 1-mm^3^ cubes, it must be noted that this virtual framework does not impose a rigid spatial scale on the internal pore network. The virtual blocks merely serve as a geometric scaffolding, providing defined planar boundaries upon which the 2-D sketches are generated. Because the dimensions and shape of these sub-volumes are inherently arbitrary, highly tortuous pore geometries and rapidly varying directional changes are effectively accommodated by manually manipulating the skinning trajectories between non-opposing faces, independent of the external bounding dimensions.

However, this labor-intensive approach was an intentional design choice. Unlike automated stochastic libraries that generate bulk statistical equivalents, deterministic spatial control over the explicit geometry of every individual sub-volume is ensured by this manual iterative optimization. Through multiple manual design cycles—where the 2-D sketches were refined and the skinning trajectories were adjusted—specific, predetermined porosity values were purposefully achieved within each individual virtual cube. This targeted approach enabled not only the construction of entirely different overall models, but also the engineering of precise, controlled spatial heterogeneity within the same model across all three spatial directions (x, y, and z).

Furthermore, the specific utilization of uniform, regular cubes combined with this localized, cube-by-cube construction inherently facilitated the precise extraction of geometric values for each of the 600 distinct sub-volumes. Consequently, a consistent statistical evaluation of spatial heterogeneity, an accurate characterization of the pore size distribution, and the establishment of a robust empirical relationship between porosity and specific surface area were permitted in the subsequent analyses.

**Figure 8:**
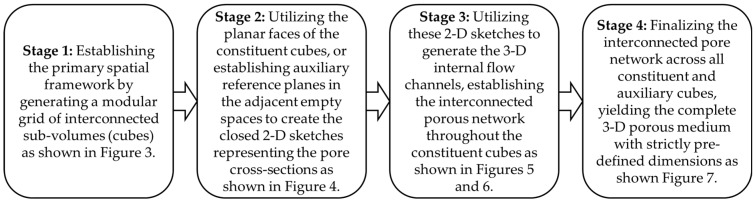
A flow diagram illustrating the sequential stages of the proposed CAD-based methodology for constructing the deterministic 3-D porous network.

It is important to emphasize that while Ansys DesignModeler was employed in this study for its seamless integration with CFD solvers, the proposed modular sketching framework is inherently software-agnostic. The geometric logic of 2-D to 3-D skinning within a virtual cube matrix can be readily executed using any standard CAD environment (e.g., SolidWorks, AutoCAD, or FreeCAD). Thus, the deterministic workflow remains accessible to the broader research community, regardless of the specific commercial or open-source platform utilized. To visually summarize this process, Figure 8 presents a comprehensive flowchart detailing the step-by-step methodology for constructing the 3-D porous networks. Furthermore, integrating parametric scripting like Python or C sharp (C#) within the CAD environment could further optimize this workflow, making it more automated and user-friendly. Such advancements would transition the manual design into a high-throughput approach for reconstructing complex reservoir architectures.

### 2.2. Creation of the Inlet Bank and the Outlet Bank with Respective Ports

**Figure 9:**
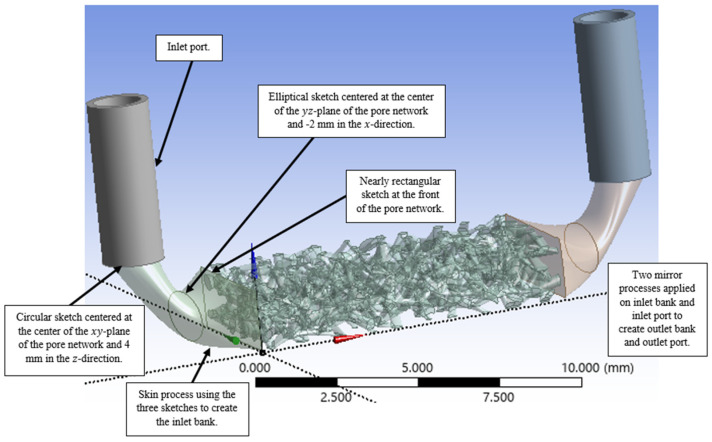
Creation of the inlet bank and the inlet port, then two mirror processes applied to create the outlet bank and the outlet port.

Following the generation of the complex porous network, the subsequent stage involves the geometric construction of the inlet and outlet ports, which includes their associated collection banks, also known as manifolds. The creation of the inlet bank is achieved first: This is accomplished through a skinning operation that utilizes three distinct profiles, or in other words, sketches. The first profile is defined at the inlet of the porous network interface. Subsequent profiles; including a distant, offset sketch and a final circular profile; are used to guide the loft. This process generates a collection bank that smoothly transitions from a horizontal orientation at the network face to a vertical orientation. Concurrently, an inlet channel (port) is created directly adjacent to this bank. Finally, this entire inlet assembly—comprising both the collection bank and the inlet channel—is symmetrically mirrored to precisely replicate the identical geometry for the outlet bank and outlet channel. Figure 9 illustrates the creation of the inlet bank and port, as well as the two mirror processes used to generate the identical outlet geometry.

### 2.3. Creation of the Main Body

**Figure 10:**
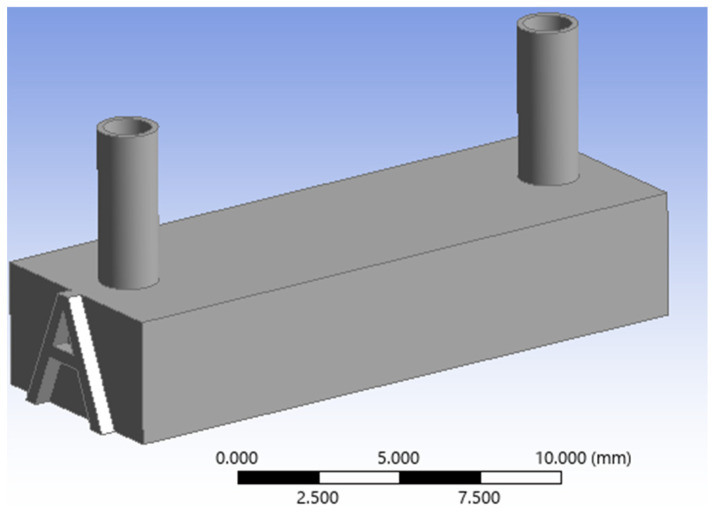
The final solid part created from the Boolean subtraction operation, showing the external rectangular prism which encases the internal fluid domain (comprising the pore network and manifolds) and features the embossed model identifier *A* on its front face.

The final stage of the geometric design was the construction of the solid main body, or housing, which fully encases the pore network assembly. To achieve this, a solid rectangular prism (cuboid) was first generated. Following the creation of this solid prism, a Boolean subtraction operation was executed. This critical operation subtracted the entire previously designed void-space geometry—which comprises the complete 3-D pore network, the inlet manifold, and the outlet manifold—from the solid prism. The result of this process is the final, single solid part, where the subtracted geometry is now precisely defined as the internal, interconnected void channels. This void space represents the complete fluid domain, ready for subsequent 3-D-printing fabrication. Finally, the corresponding model identifier (e.g., ‘*A*’, ‘*B*’, ‘*C*’) was embossed onto the front face of the solid body to ensure clear identification between the various fabricated models after 3-D printing as illustrated in Figure 10.

## 3. Results and Discussion

In this section, the main results obtained from the new computational design method are presented. A detailed analysis of the porosity and specific surface area distributions of the created models is provided.

### 3.1. Generation of Digital Micromodels and Porosity Control

**Figure 11:**
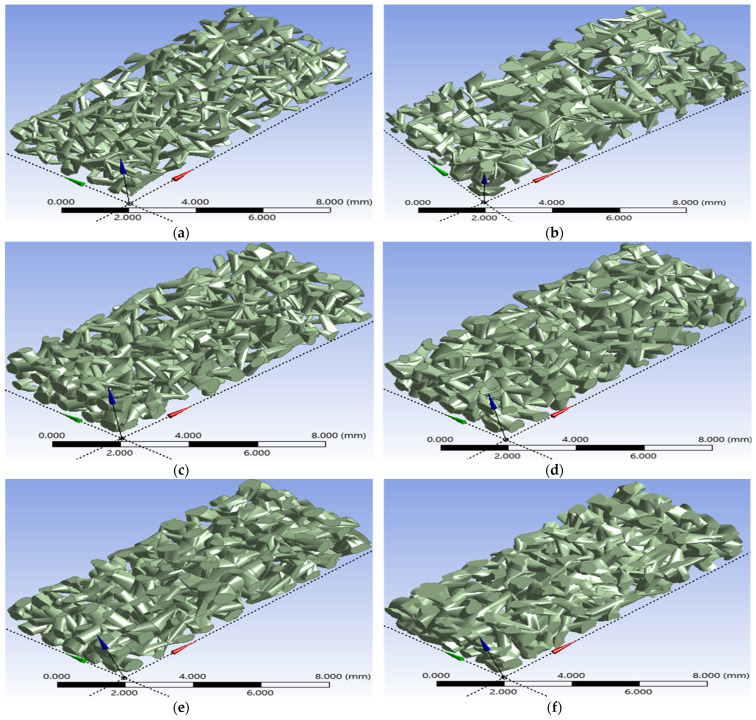
The created six channel networks with different porosities: (**a**) model *A* with a porosity of 0.184, (**b**) model *B* with a porosity of 0.226, (**c**) model *C* with a porosity of 0.286, (**d**) model *D* with a porosity of 0.341, (**e**) model *E* with a porosity of 0.393, and (**f**) model *F* with a porosity of 0.444.

The numerical methodology detailed in this study was successfully executed, leading to a validation of the design process. This implementation resulted in the generation of a suite of six distinct, three-dimensional digital micromodels, each representing a unique porous medium geometry as shown in Figure 11. These models, which are identified as models *A*, *B*, *C*, *D*, *E*, and *F*, were all systematically constructed using identical foundational virtual framework. This framework consisted of a 100-cube matrix, which established the external boundaries and the modular basis for the internal pore network generation.

This methodology, despite the uniform external volume, demonstrates control over porosity which is the primary internal network characteristic property. This capability highlights the ability of the applied method to selectively modify the internal void space while leaving the external dimensions unchanged. As illustrated in the visual results of the six networks, the generated models collectively exhibit a broad and clearly defined spectrum of porosities. This range spans from a relatively low porosity of 18.4% to a high porosity of 44.5%, covering a wide range of potential reservoir rock types.

The specific porosities achieved for each individual model were precisely quantified as follows: 18.4% for model *A*, 22.6% for model *B*, 28.6% for model *C*, 34.1% for model *D*, 39.3% for model *E*, and 44.5% for model *F*. This specific outcome serves as a critical and empirical validation of the methodology’s core strength. It provides definitive confirmation of the ability to independently control the porosity parameter. This control was achieved by systematically adjusting the geometric inputs—specifically the channel size and the number of channels created—during the 2-D loop sketch process and “skin” operations. All of this was accomplished while simultaneously keeping the fixed external dimensions of the medium.

The decoupling of the internal void geometry and the constant bulk volume represents an advancement for experimental and computational research. It effectively enables the systematic and isolated study of porosity as an independent variable, allowing researchers to investigate its specific influence on transport phenomena such as permeability or multiphase flow parameters without confusing factors from a varying sample size.

It is essential to emphasize that in characterizing these 3-D models, the specific spatial positioning of individual pore networks is considered secondary to their collective geometric attributes. Given the high density and microscopic scale of voids within natural rocks, the performance of the models is primarily dictated by its porosity, pore surface area, and length of the flow paths. By prioritizing these governing parameters, the proposed methodology ensures that the generated 3-D network accurately captures the essential physics of fluid-rock interaction and storage capacity, serving as a robust surrogate for randomly generated reservoir rock by geological conditions.

### 3.2. Geometric Validation and Morphological Analysis

To ascertain the physical realism and morphological validity of these computationally generated geometries, a detailed geometric analysis was performed on all six models. This analysis investigated the relationship between porosity (ϕ) and specific surface area (S).

It is noted that specific surface area can be defined using two primary approaches. The first, specific surface area based on bulk volume ($S_{b}$), is defined as the ratio of the surface area of the pores ($A_{s}$) to the total bulk volume ($V_{b}$):

(1) $$S_{b}=\frac{A_{s}}{V_{b}}$$

The second, specific surface area based on pore volume ($S_{p}$), is defined as the ratio of the surface area of the pores ($A_{s}$) to the pore volume ($V_{p}$) itself:

(2) $$S_{p}=\frac{A_{s}}{V_{p}}$$

These two definitions are directly linked mathematically through porosity. Dividing Equation (1) by Equation (2) yields their exact relationship:

(3) $$\frac{S_{b}}{S_{p}}=\frac{A_{s}/V_{b}}{A_{s}/V_{p}}=\frac{V_{p}}{V_{b}}=\phi$$

Equivalently:

(4) $$S_{b}=\phi S_{p}\;\mathrm{or}\;S_{p}=S_{b}/\phi$$

In this study, the empirical relationship between the bulk-volume-based specific surface area ($S_{b}$) and porosity (ϕ) was also investigated using the well-known power-law model [32,33,34,35]:

(5) $$S_{b}=C\phi^{n}$$

By substituting $S_{b}$ from Equation (4) into Equation (5), the equivalent power-law relationship for the pore-volume-based specific surface area ($S_{p}$) is obtained:

(6) $$S_{p}=C\phi^{n-1}$$

where n is the power-law index and is dimensionless, and C is the intercept on the log-log scale of $S_{b}$ (or $S_{p}$) versus ϕ. Since both ϕ and n are dimensionless, the coefficient C has the dimension of [length^−1^], which is the same dimension as $S_{b}$ (and also $S_{p}$).

**Figure 12:**
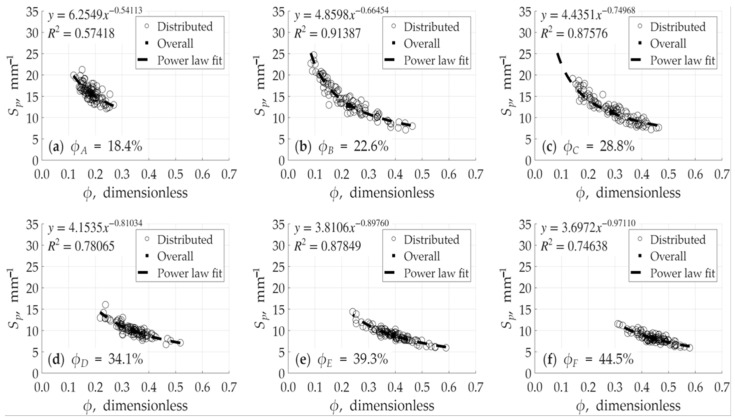
Relationship between specific surface area based on pore volume and porosity for models: (**a**) model *A*, (**b**) model *B*, (**c**) model *C*, (**d**) model *D*, (**e**) model *E*, and (**f**) model *F*. Squared black filled points represent average values of ϕ and $S_{p}$.

**Figure 13:**
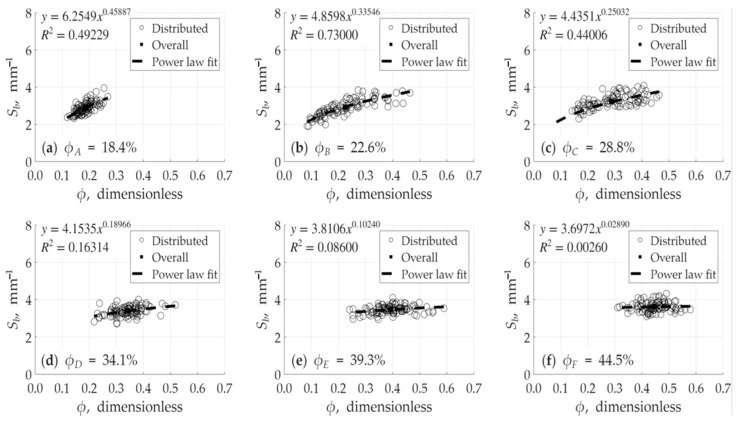
Relationship between specific surface area based on bulk volume and porosity for models: (**a**) model *A*, (**b**) model *B*, (**c**) model *C*, (**d**) model *D*, (**e**) model *E*, and (**f**) model *F*. Squared black filled points represent average values of ϕ and $S_{b}$.

The analysis revealed a relatively strong relationship between porosity (ϕ) and the specific surface area defined by pore volume ($S_{p}$) as shown in Figure 12. Conversely, the relationship between porosity (ϕ) and the specific surface area defined by the bulk volume ($S_{b}$) was found to be weaker or less direct. This behavior is illustrated Figure 13. It is also noted in these figures that the high R-squared ($R^{2}$) values, which approach 1, are a direct result of a wider porosity distribution within a single model. This wide range of local porosity values (i.e., the variation between the sub-volumes of a single model) provides a statistically robust dataset, making the underlying power-law trend clear and significant for that specific model’s internal structure. As shown in Figure 12 and Figure 13, the averaged-value points fall perfectly on the power-law curve fitting for each model.

**Figure 14:**
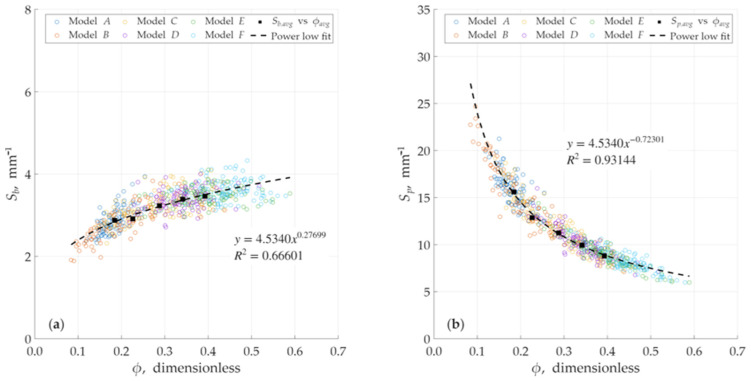
Relationship between specific surface area and porosity for models for merged data. Squared black filled points represent average values for models. (**a**) $S_{b}$ versus ϕ (**b**) $S_{p}$ versus ϕ.

Table 2 presents the overall geometric characterization data for each of the six models (model *A* through model *F*). This summary includes the average pore volume ($V_{p}$), the corresponding average porosity (ϕ), the total pore surface area ($A_{s}$), the specific surface area based on bulk volume ($S_{b}$), and the specific surface area based on pore volume ($S_{p}$) for each model individually. To illustrate the overall trend across all models, Figure 14 presents the merged data, plotting both of the specific surface area based on bulk volume ($S_{b}$) and the specific surface area based on bulk volume ($S_{p}$) separately as a function of porosity (ϕ). In this plot, the squared black filled points represent the average values for each of the six individual models. This figure visualizes the general trend for $S_{b}$ and $S_{p}$. However, this analysis confirms the primary finding that the relationship between porosity (ϕ) and the specific surface area based on pore volume ($S_{p}$) is stronger and more direct than the relationship between porosity and the specific surface area based on bulk volume ($S_{b}$), which is shown here.

This finding is expected and is clearly explained by the relationships in Equations (5) and (6). Both porosity (ϕ) and the pore-volume-based specific surface area ($S_{p}$) are fundamentally dependent on the same underlying geometric characteristics—namely, the distribution and size of the pores. Because the bulk-volume-based specific surface area ($S_{b}$) is mathematically a product of both $S_{p}$ and ϕ (as seen in Equation (4)), its direct correlation to ϕ alone is naturally less direct.

Here is an important distinction. The analysis of the relationship (shown in Figure 12 and Figure 13) is not based on only six average data points. Rather, each of the six models is comprised of 100 individual sub-volumes (the foundational cubes). This creates a comprehensive dataset of 600 data points (100 cubes × 6 models). Therefore, the high *R*-squared ($R^{2}$) values, which approach unity, are a direct result of this extensive dataset. This large population of 600 points provides a much wider and more detailed distribution of local porosity and specific surface area values. This robust statistical base allows the underlying power-law trend to be captured with high significance, confirming that the relationship is clear and not an artifact of a small sample size. Again, in Figure 14a, squared black filled points represent average values of ϕ and $S_{b}$, while in Figure 14b, squared black filled points represent average values of ϕ and $S_{p}$. These average data points fall almost perfectly on the power-law fit, which is a crucial finding. It visually corroborates the high $R^{2}$ values obtained from the full 600-point dataset. This consistency between the averaged representation and the detailed statistical analysis strongly validates the robustness of our results and confirms the accuracy of the verification. The establishment of this specific and predictable relationship is an important finding. It signifies that the generated void spaces are not simply arbitrariness, disconnected voids, but rather possess a complex, interconnected, and morphologically consistent structure. This empirical finding aligns with theoretical and experimental research on natural porous media and serves as a robust confirmation of the validity of the design methodology. The established power–law relationship in Figure 14 covers a broad and practical porosity spectrum ranging from 18% to 45%. While this interval represents a wide range for reservoir applications, the upper limit of 45% is notably high for porous media, ensuring the correlation’s reliability across diverse structural designs.

### 3.3. Pore Size Distribution

While porosity (ϕ) and specific surface area (S) provide fundamental insights into the storage capacity and fluid-solid interaction potential of the porous media, characterizing the pore size distribution is equally critical for understanding flow conductance and permeability. Since the proposed design methodology depends on generating arbitrary, non-circular closed-loop sketches on the cube faces, a direct geometric diameter documentation is not applicable. Consequently, to quantify the variation in flow channel dimensions and assess their hydraulic behavior, two equivalent diameter definitions were utilized: the hydraulic diameter ($D_{h}$) and the area-equivalent diameter ($D_{equ}$).

The hydraulic diameter ($D_{h}$) is a critical parameter for fluid dynamics calculations, particularly for estimating the Reynolds number (Re) and flow resistance in non-circular ducts. It is defined as:

(7) $$D_{h}=\frac{4A_{c}}{P}$$

where $A_{c}$ is the cross-sectional area of the channel sketch, and P is the perimeter of the sketch. Simultaneously, the area-equivalent diameter ($D_{equ}$) was calculated to represent the diameter of a circle with an equivalent area to the irregular pore shape, defined as:

(8) $$D_{equ}=\sqrt{\frac{4A_{c}}{\pi }}$$

A comprehensive statistical analysis of these geometric parameters was conducted across all generated models. Table 3 summarizes the minimum, average, and maximum values for the perimeter, the hydraulic diameter and the area-equivalent diameter. This quantitative analysis confirms that despite the arbitrariness nature of the sketch generation, the resulting pore dimensions fall within a controlled range suitable for microscale multiphase flow investigations. A significant disparity is observed between the maximum and minimum values of these geometric parameters for model *B* as detailed in Table 3. This wide range confirms the structural non-homogeneity of this model. This finding aligns perfectly with the heterogeneity analysis discussed earlier, where this model was identified as exhibiting the widest range of porosity variation, further validating the geometric consistency of the design of model.

**Figure 15:**
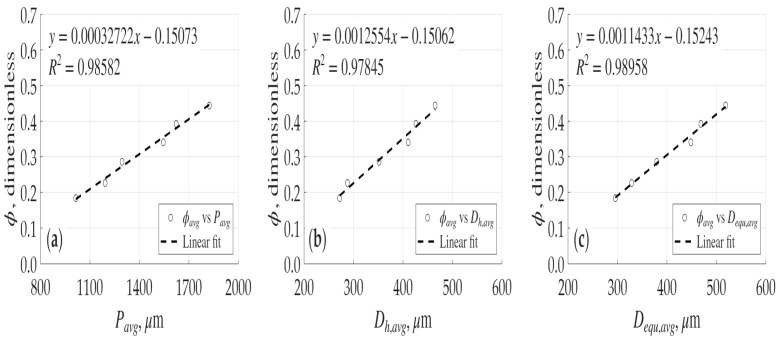
Average porosity as a function of geometric characteristics of the pore network: (**a**) versus average pore perimeter; (**b**) versus average hydraulic diameter; and (**c**) versus average hydraulic diameter.

To further validate the physical realism of the generated structures, the relationship between the average porosity and the geometric characteristics of the pore cross-sections; specifically, the average perimeter, average hydraulic diameter, and average effective diameter, was investigated. As illustrated in Figure 15, a distinct increase trend is observed where increasing porosity corresponds to an increase in all of these geometric parameters. Notably, it is observed from these figures that there are excellent linear relationships between all these parameters and the average porosity.

### 3.4. Model Applicability and Future Utility

The generated models could be used in laboratory experiments, provided that high-precision 3-D printing is available, as well as CFD. These models could be practical tools to integrate physical experimental data with numerical simulations.

#### 3.4.1. CFD Simulation and Absolute Permeability Control

**Figure 16:**
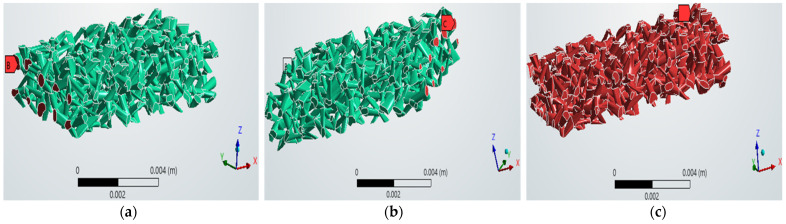
The boundary conditions defined for model *A*: (**a**) inlet boundary condition across the channels crossing the front faces from the first to tenth cube, (**b**) outlet boundary condition across the channels crossing the back faces from the ninety-first to one-hundredth cube and (**c**) all other surfaces represent wall boundary conditions.

As previously described, a primary objective of creating these porous networks is to utilize their complex boundaries as flow domains for numerical simulations. To accurately characterize the hydraulic properties and validate the decoupling of porosity and permeability, single-phase, steady-state, isothermal, laminar fluid flow was modeled using Ansys Fluent. The physical process is governed by the conservation of mass and momentum for an incompressible Newtonian fluid. The governing equations, namely the continuity and Navier–Stokes equations, are defined as:

(9) ∇·V=0

(10) $$\rho \left(V\cdot \nabla \right)V=-\nabla p+\mu \nabla^{2}V$$

where V represents the velocity vector, ρ is the fluid density, p is the pressure, and μ is the dynamic viscosity. A critical feature enabling this analysis is the explicit definition of the simulation boundaries, illustrated in Figure 16 which represents model *A*. These conditions, derived directly from the foundational 100-cube framework (Table 1), are categorized into three distinct types:

- Inlet Boundary: Positioned at the channels crossing the front faces of the first ten cubes (cubes 1–10, labeled f1–f10), this boundary serves as the entry plane for injected fluids. It can be configured as either a pressure inlet (constant pressure) or velocity inlet (fixed flow rate) to ensure uniform distribution, simulating techniques such as waterflooding (Figure 16a). In this study, a constant pressure inlet boundary condition was applied to the entry plane to determine the absolute permeability.
- Outlet Boundary: Located at channels crossing the back faces of the last ten cubes (cubes 91–100, labeled b91–b100), this boundary acts as the exit plane for produced fluids (Figure 16b). It typically operates as a pressure outlet to maintain controlled flow.
- Wall Boundary: All remaining surfaces of the channels are designated as impermeable no-flow boundaries (Figure 16c). These walls, modeled as a no-slip boundary condition, confine the fluid within the porous network domain.

By simulating the flow of synthetic brine (ρ = 1159 kg/m^3^, μ = 0.002144 Pa·s) through the digital domain, a constant pressure drop (Δp, Pa) was applied across the total length (L = 10 mm = 0.01 m). Based on the resulting average volumetric flow rate (Q, m^3^/s) and the cross-sectional area (A = 5 mm × 2 mm = 10 mm^2^ = 10^−5^ m^2^), the absolute permeability (K) was first computed in SI units (m^2^) and subsequently converted to the practical Darcy (D) unit using Darcy’s law:

(11) $$Q=\frac{KA\Delta p}{\mu L}$$

The quantitative results, summarized in Table 4, confirm the capability of the used methodology to decouple porosity from permeability. During the CFD modeling process, a nominal pressure drop of 1000 Pa was applied; however, the data presented reflects the stabilized numerical pressure drop and the resulting average volumetric flow rate for each model. To ensure the reliability of these simulations, a grid sensitivity analysis was performed. The calculated absolute permeability (K) exhibited negligible variance between the coarse and fine meshes, confirming that the numerical results are independent of mesh density.

It is important to emphasize that while the calculated permeability values (41–643 D) are inherently high due to the designed pore-scale dimensions, the primary objective of this analysis is to demonstrate the relative change and independent tunability of flow resistance through deterministic design, rather than to evaluate absolute magnitudes. Clear evidence of this control is observed in model *B*, which exhibits a significantly lower permeability than model *A* despite its higher porosity, achieved by intentionally altering the internal lofting geometries to increase tortuosity and constrict pore throats.

This precise configuration of boundary conditions and geometric determinism is critical for studying controlled fluid displacement and complex trapping mechanisms. By providing an explicit digital framework, this setup allows for the detailed analysis of phenomena such as residual oil trapping in dead-end pores (e.g., the bottom dead-end channels in cubes 1–5). Such high-fidelity representation is indispensable for accurately analyzing multiphase flow behavior, recovery efficiency, and fluid interactions. Ultimately, these results confirm that the generated domains provide a robust and validated foundation for conducting more complex numerical simulations and advanced EOR studies in hydrocarbon reservoirs.

#### 3.4.2. Linking Simulation and Physical Experimentation

A key innovation of this methodology extends beyond the digital realm. The digital designs are explicitly intended to be manufacturable. Utilizing high-resolution additive manufacturing, such as specialized 3-D printing, these digital blueprints can be transformed into physical, transparent micromodels for direct laboratory use. This capability is transformative, as it provides a direct and powerful tool for several critical applications. It allows for the direct validation of simulation results, where physical core flooding experiments can be conducted on the 3-D-printed counterparts to confirm the predictions of CFD simulations, thus creating a closed-loop validation cycle. Furthermore, like traditional micromodels, these fabricated apparatuses permit the direct visualization of internal fluid flow dynamics, a feat impossible in opaque natural core samples. This makes them cost-effective and repeatable platforms for testing and optimizing EOR processes, enabling a precise, visual understanding of fluid behavior under various injection scenarios and conditions.

#### 3.4.3. Systematic Study of Heterogeneity

Finally, the foundational 100-cube virtual framework is not merely a construction convenience; it is a tool for the systematic study of heterogeneity. Because the properties of each 1-mm^3^ cube can be controlled individually during the design phase, this methodology facilitates the controlled introduction of property variations, such as in local porosity or connectivity, both horizontally and vertically. The modular nature of the design enables the computational isolation of specific parts of the pore network for focused characterization. Any desired region can be selected while all other regions are suppressed to concentrate on this selected region.

**Figure 17:**
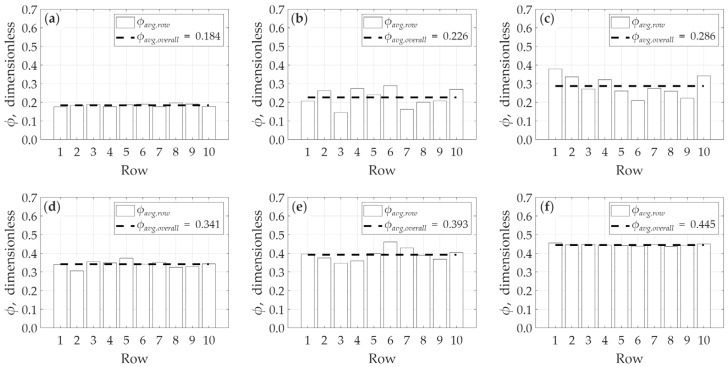
Horizontal heterogeneity in porosity in x-direction: (**a**) model *A*, (**b**) model *B*, (**c**) model *C*, (**d**) model *D*, (**e**) model *E*, and (**f**) model *F*.

Figure 17 illustrates the horizontal variation in porosity along the x-direction. This data is presented by averaging the porosity for each of the ten constituent block rows (e.g., Row 1 represents the average porosity for cubes 1–10, Row 2 represents the average for cubes 11–20, and so on, up to the tenth row). From this figure, the heterogeneous nature of the models is evident. Model *B* is observed to be the most heterogeneous, showing the widest range of porosity variation between the rows, followed by model *C*. Conversely, model *A* and model *F* are identified as the most homogeneous, exhibiting the least range of variation along the x-direction. This observation directly supports the statistical analysis of the internal data presented in Figure 12. The models identified here as the most heterogeneous (like *B* and *C*) possess a wider internal distribution of local porosity values; this wide statistical range is precisely what yields a high R-squared ($R^{2}$) value approaching unity. In contrast, the most homogeneous models (*A* and *F*) have a comparatively narrower internal porosity distribution, which corresponds to their relatively lower (though still significant) R-squared values.

**Figure 18:**
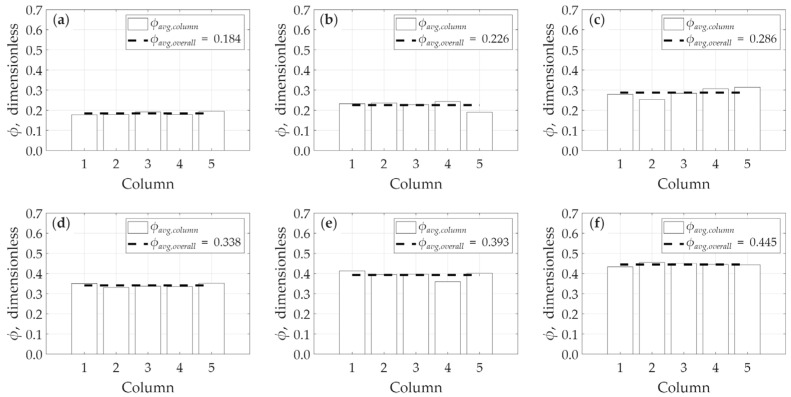
Horizontal heterogeneity in porosity in y-direction: (**a**) model *A*, (**b**) model *B*, (**c**) model *C*, (**d**) model *D*, (**e**) model *E*, and (**f**) model *F*.

In a similar manner, Figure 18 presents the analysis of porosity variation along the y-direction. This plot shows the average porosity for each of the five constituent columns; each of these column averages is calculated from a sample of 20 cubes. Despite the same trend in heterogeneity in y-direction as well as in x-direction, a key observation is that the apparent heterogeneity (the variation between these column averages) appears significantly lower in this y-direction analysis compared to the variation observed in the x-direction (Figure 17). This reduction in apparent variation is an expected statistical outcome. The sample size used for averaging in the y-direction (N = 20 cubes) is exactly double the sample size used for the x-direction analysis (N = 10 cubes). Statistically, as the sample size for an average increase, the variance of the sample means decreases (in line with the Central Limit Theorem). This causes the calculated averages to cluster more tightly around the overall mean, resulting in a ‘smoothing’ effect that makes the heterogeneity less visually apparent than in the x-direction analysis.

**Figure 19:**
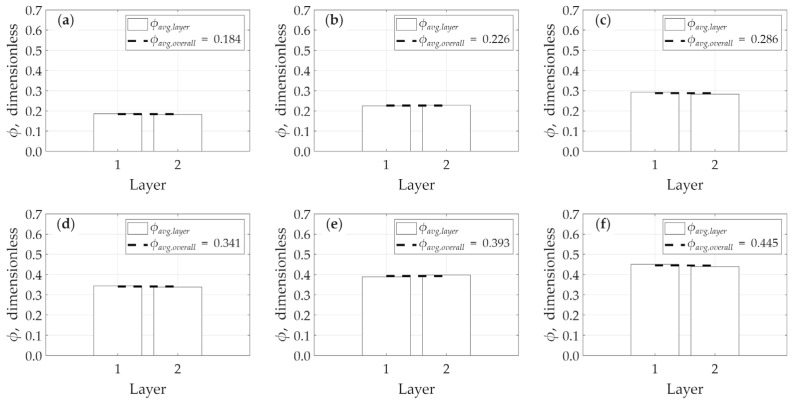
Vertical heterogeneity in porosity in z-direction: (**a**) model *A*, (**b**) model *B*, (**c**) model *C*, (**d**) model *D*, (**e**) *E*, and (**f**) model *F*.

This statistical smoothing effect becomes even more pronounced when analyzing the vertical porosity variation along the z-direction as illustrated in Figure 19. In this analysis, the model is divided into only two layers: the bottom layer, representing the average porosity of the lower 50 cubes, and the top layer, representing the average porosity of the upper 50 cubes. Due to this very large sample size (N = 50) used for each average, all models appear to be almost perfectly homogeneous in the vertical direction, as the variations between the two layers are statistically minimized.

## 4. Conclusions

This study presents a novel computational methodology for designing 3-D micromodels of porous media that simulate hydrocarbon reservoirs. The method has proven highly effective, generating six digital models with a consistent bulk volume (100 mm^3^) while exhibiting a wide range of porosities from 18.4% to 44.4%. A key strength of this approach is the ability to independently control porosity, by adjusting channel size and number, and permeability, by modifying the degree of pore connectivity.

This methodology facilitates the systematic study of heterogeneity. As the model is constructed from a virtual framework of 100 cubes, property variations can be introduced horizontally and vertically. Furthermore, the design process, which involves suppressing the solid cubes, allows for the selection of allowing the selection specific parts of the pore network for focused characterization while isolating or hiding other sections.

Crucially, unlike stochastic algorithmic methods that rely on bulk statistical approximations, this deterministic approach provided explicit geometric control over individual sub-volumes. This unique capability permitted a systematic investigation of spatial heterogeneity across all three spatial directions (x, y, and z). Consequently, it facilitated the precise extraction of pore volume distributions and the establishment of a direct correlation between localized porosity and specific surface area based on exact geometric data rather than random statistical generation.

To validate the generated geometries, the analysis revealed a distinct power-law relationship between porosity and specific surface area. This finding is significant as it aligns with previously published research, thereby confirming the validity of the design methodology.

In addition to analysis, the pore size distribution was characterized by calculating the perimeter, hydraulic diameter, and area-equivalent diameter of the pore cross-sections. This analysis revealed strong linear relationships between these geometric parameters and average porosity, confirming that an increase in increase in pore dimensions corresponds to a consistent and predictable increase in porosity.

The primary advantage of this work is the creation of a digital domain that accurately mimics tortuous fluid paths, making the models ideal for advanced CFD simulations. Single-phase numerical simulations confirmed the capability to independently tune and decouple absolute permeability from porosity. By adjusting internal tortuosity and pore-throat constriction, model *B* exhibited a lower permeability than model *A* despite its higher porosity.

While the current study focuses on foundational design and geometric validation, these digital models are inherently manufacturable via 3-D printing. As a planned future application, these physical models will provide a powerful tool to bridge the gap between advanced numerical simulations and physical laboratory experiments. This future validation phase will facilitate a precise understanding of pore-scale fluid behavior, ultimately aiding in the optimization of EOR processes.

Finally, a detailed heterogeneity analysis was conducted in all directions. The horizontal analysis in the *x*-direction, for instance, confirmed that models with a wider internal distribution of local porosity values (i.e., greater heterogeneity) correspondingly yielded a stronger statistical correlation ($R^{2}$ approaching unity) for the relationship between specific surface area based on pore volume and porosity. This finding highlights the robustness of the data generated from the more heterogeneous models.

## Figures and Tables

**Table 1:** The faces of the first five cubes and the last five cubes.

Cube	Created or Imaginary	Origin of Front-Right-Bottom Corner	Front	Back	Right	Left	Bottom	Top
1	Created	(0, 0, 0)	*f*_1_	*b*_1_ *f*_11_	*r*_1_	*l*_1_ *r*_2_	*bo*_1_	*t*_1_ *bo*_6_
2	Imaginary	(0, 1 mm, 0)	*f*_2_	*b*_2_ *f*_12_	*l*_1_ *r*_2_	*l*_2_ *r*_3_	*bo*_2_	*t*_2_ *bo*_7_
3	Created	(0, 2 mm, 0)	*f*_3_	*b*_3_ *f*_13_	*l*_2_ *r*_3_	*l*_3_ *r*_4_	*bo*_3_	*t*_3_ *bo*_8_
4	Imaginary	(0, 3 mm, 0)	*f*_4_	*b*_4_ *f*_14_	*l*_3_ *r*_4_	*l*_4_ *r*_5_	*bo*_4_	*t*_4_ *bo*_9_
5	Created	(0, 4 mm, 0)	*f*_5_	*b*_5_ *f*_15_	*l*_4_ *r*_5_	*l*_5_	*bo*_5_	*t*_5_ *bo*_10_
: :		: :	: :	: :	: :	: :	: :	: :
96	Created	(9 mm, 0, 1 mm)	*b*_86_ *f*_96_	*b*_96_	*r*_96_	*l*_96_ *r*_97_	*t*_91_ *bo*_96_	*t*_96_
97	Imaginary	(9 mm, 1 mm, 1 mm)	*b*_87_ *f*_97_	*b*_97_	*l*_96_ *r*_97_	*l*_97_ *r*_98_	*t*_92_ *bo*_97_	*t*_97_
98	Created	(9 mm, 2 mm, 1 mm)	*b*_88_ *f*_98_	*b*_98_	*l*_97_ *r*_98_	*l*_98_ *r*_99_	*t*_93_ *bo*_98_	*t*_98_
99	Imaginary	(9 mm, 3 mm, 1 mm)	*b*_89_ *f*_99_	*b*_99_	*l*_98_ *r*_99_	*l*_99_ *r*_100_	*t*_94_ *bo*_99_	*t*_99_
100	Created	(9 mm, 4 mm, 1 mm)	*b*_90_ *f*_100_	*b*_100_	*l*_99_ *r*_100_	*l*_100_	*t*_95_ *bo*_100_	*t*_100_

**Table 2:** Overall characterization for all models.

Model	Pore Volume, mm^3^	Porosity, Fraction	Pore Surface Area, mm^2^	Specific Surface Area Based on Bulk Volume, mm^−1^	Specific Surface Area Based on Pore Volume, mm^−1^
*A*	18.4	0.184	288	2.88	15.6
*B*	22.6	0.226	291	2.91	12.8
*C*	28.6	0.286	323	3.23	11.3
*D*	34.1	0.341	339	3.39	9.94
*E*	39.3	0.393	346	3.46	8.82
*F*	44.5	0.444	362	3.62	8.15

**Table 3:** Statistical summary of perimeter, hydraulic diameter and area-equivalent diameters for all models.

Model	Perimeter, μm			Hydraulic Diameter, μm			Area-Equivalent Diameter, μm		
	Minimum	Maximum	Average	Minimum	Maximum	Average	Minimum	Maximum	Average
A	320	1984	1014	88	608	272	95	599	296
B	56	3149	1192	18	706	288	18	773	329
C	435	2698	1294	124	681	351	131	709	379
D	790	2530	1545	223	704	410	247	728	448
E	716	2671	1624	188	751	426	207	799	468
F	862	3072	1824	242	839	465	274	884	519

**Table 4:** Numerical determination of absolute permeability for models A–F under a nominal applied pressure drop of 1000 Pa.

Model	Mesh	Stabilized Numerical Applied Pressure Drop, Pa	Average Volumetric Flow Rate, m^3^/s	Absolute Permeability, D
*A*	Coarse	998.8	3.97 × 10^−8^	86.3
	Fine	998.9	3.85 × 10^−8^	83.8
*B*	Coarse	999.8	1.91 × 10^−8^	41.6
	Fine	999.9	1.90 × 10^−8^	41.2
*C*	Coarse	999.4	7.08 × 10^−8^	155
	Fine	999.4	7.12 × 10^−8^	155
*D*	Coarse	996.9	1.54 × 10^−7^	338
	Fine	997.0	1.55 × 10^−7^	338
*E*	Coarse	994.3	2.62 × 10^−7^	572
	Fine	994.4	2.60 × 10^−7^	570
*F*	Coarse	989.6	2.93 × 10^−7^	644
	Fine	989.7	2.93 × 10^−7^	643

## Data Availability

The data presented in this study are available on request from the corresponding author.

## Abbreviations

2-D	Two-dimensional
3-D	Three-dimensional
*b*	Back
*bo*	Bottom
BPR	Back Pressure Regulator
CAD	Computer-Aided Design
CFD	Computational Fluid Dynamics
CT	Computerized Tomography
C#	C sharp language
D	Darcy unit
EOR	Enhanced Oil Recovery
*f*	Front
kg	Kilogram
*l*	Left
m	Meter
mm	Millimeter
Pa	Pascal
PDMS	Polydimethylsiloxane
*r*	Right
RS	Representative Scale
s	Second
SI units	International System of Units (Système International d’unités)
*t*	Top

## Nomenclature

*A*, *B*, *C*, *D*, *E* and *F*	Model codes
A	Cross-sectional area of flow domain, m^2^
$A_{c}$	Cross-sectional area, mm^2^
$A_{s}$	Surface area of the pores, mm^2^
C	Intercept on the log-log scale, mm^−1^
$D_{equ}$	Area-equivalent diameter, μm
$D_{equ,avg}$	Average area-equivalent diameter, μm
$D_{h}$	Hydraulic diameter, μm
$D_{h,avg}$	Average hydraulic diameter, μm
i , j and k	Unit vectors in x-, y-and z-directions, respectively, dimensionless
K	Absolute permeability, m^2^ and D
L	Length of flow domain, m
n	Power-law index, dimensionless
*N*	Sample size
P	Perimeter, μm
p	Pressure, Pa
$P_{avg}$	Average perimeter, μm
Q	Volume flow rate, m^3^/s
R	Correlation coefficient, dimensionless
$R^{2}$	Correlation coefficient squared, dimensionless
Re	Reynolds number
S	Specific surface area, mm^−1^
$S_{b}$	Specific surface area based on bulk volume, mm^−1^
$S_{b,avg}$	Average specific surface area based on bulk volume, mm^−1^
$S_{p}$	Specific surface area based on pore volume, mm^−1^
$S_{p,avg}$	Average specific surface area based on pore volume, mm^−1^
V	Velocity vector = ui+ vj+ wk for cartesian coordinates, m/s
$V_{b}$	Bulk volume, mm^3^
$V_{p}$	Pore volume, mm^3^
u , v and w	Velocity components in x-, y-and z-directions, respectively, m/s
x , y and z	Coordinates in cartesian coordinates, mm
Δ	Drop or change
μm	Micrometer
μ	Absolute or dynamic viscosity, Pa.s
π	Pi ≈ 3.1416
ρ	Density, kg/m^3^
ϕ	Porosity, dimensionless
$\phi_{avg}$	Average porosity, dimensionless
$\phi_{avg,column}$	Column average porosity, dimensionless
$\phi_{avg,layer}$	Layer average porosity, dimensionless
$\phi_{avg,overall}$	Model average porosity, dimensionless
$\phi_{avg,row}$	Row average porosity, dimensionless
∇	$\mathrm{Nabla}\;\mathrm{operator}\;=\left(\partial /\partial x\right)\;i+\;\left(\partial /\partial y\right)j+\;\left(\partial /\partial z\right)k$ for cartesian coordinates, 1/m
·	Dot product operators of two vectors
